# Socioeconomic status, loneliness, and depression among older adults: a cross-sectional study in Spain

**DOI:** 10.1186/s12877-024-04978-3

**Published:** 2024-04-23

**Authors:** Esteban Sánchez-Moreno, Lorena Gallardo-Peralta, Ana Barrón López de Roda, Joaquín M. Rivera Álvarez

**Affiliations:** 1 Department of Sociology: Methods and Theory, Universidad Complutense de Madrid, Madrid, Spain; 2 Department of Social Work and Social Services, Faculty of Social Work, Universidad Complutense de Madrid, Campus de Somosaguas, 28223 Pozuelo, Madrid, Spain; 3 Department of Social, Organizational and Differential Psychology, Universidad Complutense de Madrid, Madrid, Spain; 4 Faculty of Social Work, Universidad Complutense de Madrid, Madrid, Spain

**Keywords:** Social loneliness, Emotional loneliness, Mental health, Social determinants of health, Education, Economic hardships, Living arrangements, Nursing homes

## Abstract

**Background:**

The association between socioeconomic status and depression is weaker in older adults than in younger populations. Loneliness may play a significant role in this relationship, explaining (at least partially) the attenuation of the social gradient in depression. The current study examined the relationship between socioeconomic status and depression and whether the association was affected by loneliness.

**Methods:**

A cross-sectional design involving dwelling and nursing homes residents was used. A total of 887 Spanish residents aged over 64 years took part in the study. Measures of Depression (GDS-5 Scale), Loneliness (De Jong-Gierveld Loneliness Scale), Socioeconomic Status (Education and Economic Hardship), and sociodemographic parameters were used. The study employed bivariate association tests (chi-square and Pearson’s r) and logistic regression analyses.

**Results:**

The percentage of participants at risk of suffering depression was significantly higher among those who had not completed primary education (45.5%) and significantly lower among those with university qualifications (16.4%) (X^2^ = 40.25;*p* <.001), and respondents who could not make ends meet in financial terms faced a higher risk of depression (X^2^ = 23.62;*p* <.001). In terms of the respondents who experienced loneliness, 57.5% were at risk of depression, compared to 19% of those who did not report loneliness (X^2^ = 120.04;*p* <.001). The logistic regression analyses showed that having university qualifications meant a 47% reduction in the risk of depression. This risk was 86% higher among respondents experiencing financial difficulties. However, when scores for the loneliness measure were incorporated, the coefficients relating to education and economic hardships ceased to be significant or were significantly reduced.

**Conclusion:**

Loneliness can contribute to explaining the role played by socioeconomic inequalities in depression among older adults.

## Introduction

Depression is a key issue during ageing. In Europe, depression is more prevalent among people aged over 64 years than among other age groups [1]. Depression decreases quality of life for older adults [2] and increases the risk of deterioration of health and functionality [3, 4]. The existing literature has confirmed the significance of socioeconomic status (SES) for mental health among older adults. Specifically, the empirical evidence indicates the presence of a socioeconomic gradient in the case of depression [5]. There are two main indicators for SES in the literature on health: education and income [6]. Several studies have shown that education is linked to a lower prevalence of depression [7] and can be a significant protective factor [8]. However, other studies have not identified a significant association between education and depressive symptoms [9] and some studies have even reported higher scores for depression among those with higher levels of education [10]. In short, as noted by Li and Zhao [11], the empirical evidence on the relationship between education and depression in later life is inconclusive.

The literature on ageing also reports an inconclusive relationship between income and depression, with some studies finding a significant association and others not [12–14]. In fact, as with education, the association between income and depression is heavily influenced by contextual variables (State, national) [5] related to policies for the protection of older adults, and particularly pension and health systems. As stated in these studies [15], it can be difficult to use income as a measure for SES in the case of older adults given that in a majority of cases this income derives from standards established by retirement systems following the cessation of employment. In this regard, economic hardship would be a more accurate approach to examine the role that income plays in psychological wellbeing among older adults, and particularly as regards depressive symptoms. The concept of economic hardship describes a situation where someone has insufficient income to cover their daily socioeconomic needs. This process acts as a stressor for individuals (financial strain) and threatens their ability to meet basic needs. Its relationship with depression has been reported in numerous studies [16–19]. More specifically, the existing research underlines the role played by recent economic hardship, which has a greater impact on depressive symptoms than difficulties experienced in the more distant past [20]. Finally, it is worth noting the work of Sun et al. [21], who found that financial stress continues to affect mental health even when controlled by household income, which suggests that its potential effect may be only partly related to income.

In summary, an analysis of the published empirical evidence shows that the SES gradient for depression among older adults is characterised by specific elements compared to other age groups. In fact, several articles suggest that inequalities of this kind are reduced in the case of older adults. In other words, there are processes that are characteristic of ageing and reduce the social gradient in health, meaning that SES might not be such a significant determinant compared to its impact among the younger or middle-aged population [22]. This hypothesis, which can be defined as the age-as-leveller hypothesis, suggests that socioeconomic differences in health increase during adulthood and accumulate to affect health during middle age. However, the following factors come into play at older ages: (a) biological processes leading to increased fragility; and (b) social, economic and health policies specifically aimed at older people. Both processes contribute to reducing the effects of low SES on health, causing a weakening of the education and income gradients with older age [23].

The impact of psychosocial processes closely linked to ageing also has to be taken into account in the case of mental health, and particularly depression. In this paper, we propose that loneliness is one of these factors. In this regard, the aim of this research was to analyse the relationship between socioeconomic status and depression and whether the association was affected by loneliness. The starting point was to consider loneliness as a key process to understand the role played by SES in depression among older adults. With good reason, the study of loneliness has recently become one of the key lines of research regarding the wellbeing of older adults [24]. The existing evidence had already demonstrated the importance of relationships and social support for depression among older people [25]. This evidence showed that functional aspects of social support from various sources act as buffers against negative life experiences, thereby reducing the potential of such experiences to cause depression [26]. The literature examining loneliness has focused on this line of study. The ageing process is characterised by the narrowing of support networks and a potential increase in situations of loneliness [27]. Loneliness is an unpleasant experience that occurs when social relationships are insufficient in quantity and quality [28]. Along these lines, de Jong-Gierveld [29, p.73–74] defined loneliness as “a situation in which the number of existing relationships is smaller than is considered desirable, as well as situations where the intimacy one wishes for has not been realized”. Various authors have described the multidimensional nature of this concept [30]. Emotional and social loneliness are frequently distinguished in this regard. The former arises from a lack of the emotional support provided by close relationships, while the latter occurs in the absence of an adequate social network. The available empirical evidence has shown that loneliness is a risk factor for the development of depressive symptoms among older people [31, 32]. As part of their systematic review, Lambert Van As et al. [33] concluded that the published literature shows the existence of both a longitudinal association between loneliness and depressive symptoms and an unfavourable course of depression associated with loneliness.

As previously stated, the aim of this research was to analyse the role that loneliness plays in the relationship between SES and depression. The findings of recent studies suggest that loneliness and SES are inter-related processes, with objective financial measures of financial strain (such as income, financial downturn and income disparities) associated with higher levels of loneliness [34–36]. Specifically, subjective measures of financial strain are associated with higher levels of loneliness, and this association could be even more intense than the link with objective measures of economic hardship [37]. There is contradictory empirical evidence in the case of education. Some studies suggest that loneliness is more intense and/or frequent among people with lower levels of education [38–40], but others have found no association [41, 42] and there are even studies whose findings suggest that higher qualifications are associated with greater experiences of loneliness [43].

It hence appears important to empirically analyse the relationship between SES and depression, taking into account the role that loneliness plays in that relationship. More specifically, this study was based on the following particular aims: (a) to analyse the association between SES and experiences of loneliness; (b) to analyse the association between SES and depression; (c) to identify changes to the pattern of association between SES and depression as a result of the incorporation of (emotional and social) loneliness into the analyses; and (d) to explore the existence of interaction between SES and loneliness in the case of depression.

## Materials and methods

### Participants and procedure

A total of 887 Spanish residents took part in the study. The inclusion criteria were as follows: (a) aged at least 65 years; (b) living with partner (no other people living at home) or living alone, or residing in a nursing home for more than six months; (c) no severe cognitive impairment; and (d) able to communicate. For community-dwelling participants, the procedure included the application of the Mini-Mental State Examination test only in cases where signs of severe cognitive impairment were detected (mild cognitive impairment was not an exclusion criterion) at the time of informing participants about the study objectives and requesting their consent. On the other hand, the researchers collaborated with the professionals of the nursing homes to identify participants who met the inclusion criteria for the study. The participants’ mean age was M = 78.5, SD = 8.7, and 62% of participants were women.

The data were collected using a survey administered by trained personnel. Following the initial contact with potential participants and once their informed consent had been obtained, the surveys were applied under the conditions chosen by those who agreed to take part. For nursing home residents, the surveys were applied in an area of their nursing home that ensured the interview would be confidential. All study procedures were approved by the Ethics Review Board of the Universidad Complutense (Madrid) (report reference CE_20220217-14_SOC).

### Measures

#### Outcome variable

Depression. The five-item version of the Geriatric Depression Scale (GDS-5) was used. The short version of the Geriatric Depression Scale maintains the effectiveness of the original scale while improving ease of administration [44]. This instrument is used to record the presence of five depressive symptoms, producing a score ranging from 0 to 5. The validation of the five-item version in Spain [45] showed adequate sensitivity levels that are comparable to those of the two longer versions. The cut-off point of ≥2, recommended by Hoyl et al. [44] was used for the identification of potential cases. The Cronbach’s Alpha coefficient was 0.701.

#### Exposure variables

Loneliness. The six-item version of the De Jong Gierveld loneliness scale [46] (DJGLS) was used. The DJGLS scale was validated in Spain by Ayala et al. [47] for a sample of older adults. It is a short and commonly used instrument in loneliness research that produces an overall score and two sub-scores for social and emotional loneliness. This multidimensional approach makes this instrument the most appropriate one to achieve the aims of this research. Items are scored on a scale from 0 to 2, although they are subsequently recodified as dichotomous (0 or 1). The scale include items that required reverse scoring, such as ‘There are many people I can trust completely.’ The recoding process ensured consistency in the direction of responses. Higher scores on the total scale indicate stronger feelings of loneliness (range from 0 to 6). The loneliness scale was used in two ways for the analyses on which this article is based. First, the total scores for both subscales (emotional and social loneliness) were used as predictive variables for the outcome variable values. Second, participants with an overall DJGLS score of 3 or higher were classified as being in situations involving a risk of loneliness. Typically, three cut-off points are used for this scale (0–1: not lonely; 2–4: moderately lonely; 5–6: strongly lonely). However, de Jong-Gierveld and Van Tilburg [48, p.12] point out that “there is the problem that the scale for emotional loneliness is more closely related to the direct question of loneliness than the scale for social loneliness. It also applies that an equal cut-off point for both subscales does not result in a similar score on the shortened scale”. In the present study, the cut-off point to identify a situation of risk of loneliness (versus no risk) was established, following the strategy of Rodríguez-Blázquez et al. [49]. The analyses were carried out separately to avoid collinearity risks. The Cronbach’s Alpha coefficient was 0.751.

Socioeconomic status (SES). Two indicators were selected to measure socioeconomic status. First of all, level of education was used given its recognised value as an indicator of SES in the literature on social determinants of health [6]. Education was classified into four large categories in this study: incomplete primary education (including unqualified people); completed primary education; secondary education; and university education. Economic hardship was used as the second indicator: the presence of difficulties making ends meet in financial terms. This indicator is frequently used to indirectly measure limitations arising from income levels. The following specific item was used: “Regardless of your household income and taking into account your total monthly income, would you say you can make ends meet?”. Four response categories were offered: (1) easily; (2) reasonably easily; (3) with some difficulty; and (4) with great difficulty. Categories 1 and 2 were combined, as were categories 3 and 4, to produce a dichotomous variable (1 = difficulties making ends meet).

#### Control variables

The variables of sex (1 = female), age, children (1 = no children) and limitations on activities of daily living due to health problems were incorporated. The latter variable was evaluated using the Global Activity Limitation Indicator (GALI) [50]. The GALI consists of a single item (“For at least the last six months, have you been limited because of a health problem in activities people usually do?”), with three response categories (yes, strongly limited; yes, limited; no, not limited). The GALI is a useful and valid instrument to assess global activity limitation in both health and non-health surveys.

Living arrangement. This was used as a control variable in models that included loneliness among the predictors of the outcome variable. This decision was based on the available empirical evidence regarding the notable impact of the three situations taken into account in this study (living with partner only; living alone; living in a nursing home) on levels of loneliness experienced by older adults when compared with other living arrangements [51, 52].

### Analysis

First, the descriptive statistics of the study variables and bivariate association statistics were obtained. Second, a logistic regression analysis was performed to test the fit of five models. The base model only incorporated the control variables. The SES indicators were then included in the equation (model 2: “Socioeconomic Status”). The third model (“Loneliness”) included the dimensions of loneliness considered in the study. Models 4 and 5 successively incorporated interactions between the variables related to SES and loneliness. Third, the logistic regression equations for models 3 to 5 were calculated by replacing the scores for the two dimensions of loneliness (emotional and social) with a variable estimating the existence of a risk of loneliness based on a cut-off point (as described in the previous section).

## Results

Table 1 shows the distribution of the main study variables. The high prevalence of depression and loneliness is due to the sample composition. In fact, the present study included participants living alone, with their partner, or in a nursing home, precisely because such living arrangements are especially relevant to the study of loneliness. In this sense, it was necessary to ensure the presence of a sufficient number in the sample of each situation, especially in the case of people living in a residence (20.7% of the participants). Table 1 disaggregates the information according to living arrangements. As expected, the percentage of people with limitations in ADL due to health problems is higher among those who live in a nursing home. The percentage of women and childless people is also higher. On the other hand, economic hardships are less frequent in this group. Table 2 shows the descriptive statistics and correlation coefficients for the quantitative study variables. As may be observed, all coefficients are significant except for that measuring the association between age and social loneliness.

**Table 1:** Participants’ depression, social and demographic characteristics

Variable	Total	Living alone	Living with partner	Nursing home
Depression (risk, cut-off point = 2)	29.9%	29.5%	18.1%	54.3%
Loneliness (risk, cut-off point = 3)	26.2%	29.1%	15.4%	44.5%
Women	62.1%	66.3%	53.4%	72.3%
Have children	80.6%	76.2%	90.6%	68.5%
*Limitation on ADL*				
Not limited	54.1%	53.5%	62.3%	39.8%
Limited	34.5%	38.4%	29.9%	37.6%
Strongly limited	10.9%	8.1%	7.8%	22.6%
*Education*				
Incomplete primary education	23.7%	25.5%	15.6%	37.0%
Complete primary education	44.8%	45.5%	46.4%	40.3%
Secondary	15.0%	15.7%	16.2%	11.1%
University	16.6%	13.3%	21.8%	11.6%
Economic harship	26.7%	28.6%	27.9%	19.6%
*Living arrangement*				
Living alone	37.4%	-	-	-
Living with partner only	41.8%	-	-	-
Nursing home	20.7%	-	-	-

**Table 2:** Mean (standard deviation) and Pearson’s correlation coefficients for key study variables

	Age	Loneliness score	Depression score	Emotional loneliness	Mean (SD)
Age	-				78.5 (8.7)
Loneliness (score)	0.100**	-			1.63 (1.69)
Depression (score)	0.181**	0.556**	-		1.03 (1.3)
Emotional loneliness	0.140**	0.802**	0.568**	-	0.84 (0.92)
Social loneliness	0.040	0.867**	0.379**	0.398**	0.79 (1.1)

^***^p <.001; ^**^p <.01; ^*^p <.05

The results for the bivariate analyses referring to qualitative variables (see Table 3) showed that the risk of being above the GDS-5 cut-off point (for depression) was higher for women (χ^2^ = 10.45;*p* <.001) and lower for those who did not suffer limited mobility owing to illness (χ^2^ = 106.34;*p* <.001) than for those who faced mild or serious limitations. In addition, the percentage of participants at risk of depression was significantly higher among those who had not completed primary education (45.5%) and significantly lower among those who had obtained university qualifications (16.4%) (χ^2^ = 40.25;*p* <.001). Meanwhile, participants who had difficulty making ends meet faced a higher risk of depression (X^2^ = 23.62;*p* <.001). Residential status was also significantly associated with the risk of depression (χ^2^ = 77.34;*p* <.001). Specifically, 54.3% of people in nursing homes and 29.5% of those living alone were above the established cut-off point, compared to 18.1% of those living with a partner. Notably, 57.5% of those experiencing loneliness were at risk of depression, as opposed to 19% of those who were not experiencing loneliness (χ^2^ = 120.04;*p* <.001).

**Table 3:** Bivariate analysis between social and demographic variables and depression and loneliness

Variable	Depression (risk, cut-off point = 2)	χ^2^; p	Loneliness (risk, cut-off point = 3)	χ^2^; p
***Sex***				
Women	23.5%	χ^2^ = 10.46 *p* =.001	25.9%	χ^2^ = 0.018 *p* =.892
Men	33.8%		26.3%	
***Children***				
Have children	29.2%	χ^2^ = 0.73 *p* =.392	23.1%	χ^2^ = 17.77 *p <*.001
Do not have children	32.6%		39.3%	
***Limitation on ADL***				
Not limited	16.0%	χ^2^ = 106.34 *p <*.001	17.6%	χ^2^ = 42.24 *p <*.001
Limited	41.8%		33.6%	
Strongly limited	59.8%		44.2%	
***Education***				
Incomplete primary education	45.5%	χ^2^ = 40.25 *p <*.001	38.4%	χ^2^ = 26.91 *p <*.001
Complete primary education	28.9%		26.2%	
Secondary	22.7%		20.0%	
University	16.4%		15.2%	
***Economic harship***				
Yes	41.2%	χ^2^ = 23.62 *p <*.001	39.9%	χ^2^ = 34.85 *p <*.001
No	23.9%		19.6%	
***Living arrangement***				
Living alone	29.5%	χ^2^ = 77.34 *p <*.001	29.1%	χ^2^ = 51.95 *p <*.001
Living with partner only	18.1%		15.4%	
Nursing home	54.3%		44.5%	

Bivariate analyses were conducted for loneliness and the other variables (Table 3), due to the importance of loneliness for this study. The findings showed no differences between men and women, with experiences of loneliness more common among participants who did not have children (χ^2^ = 17.77;*p* <.001) and those who faced limitations affecting their daily activities (χ^2^ = 42.24;*p* <.001). The percentage of people experiencing loneliness was significantly lower among participants with university qualifications (15.2%) and significantly higher (38.4%) among unqualified participants (χ^2^ = 10.45;*p* <.001). Strikingly, 39.9% of people with difficulties making ends meet experienced loneliness, compared to 19.6% in the case of people who did not face economic hardship (χ^2^ = 34.84;*p* <.001). Finally, 44.5% of participants living in nursing homes and 29.1% of those living alone were above the cut-off point for loneliness. This percentage fell to 15.4% among those living with a partner (χ^2^ = 51.95;*p* <.001).

Table 4 summarises the results obtained from the logistic regression analysis incorporating the two dimensions of loneliness (emotional and social) as quantitative variables. As may be observed, in model 2 the variables related to socioeconomic status had a statistically significant association with the outcome variable, such that having university qualifications implied a 47% reduction in the likelihood of depression. In contrast, the likelihood of depression was 86% higher among those experiencing economic hardship. Table 4 demonstrates the importance of living arrangements, with the potential for depression being 103% higher among those living in nursing homes. However, model 3 represents a notable shift in the range of variables significantly associated with the depression measure (GDS-5). This model incorporated the two dimensions of loneliness considered in the study: emotional and social. Of these two, only the former showed a positive and statistically significant association with the outcome variable, meaning that the likelihood of identifying a case of depression increased in the presence of emotional loneliness. The inclusion of social loneliness in the model did not result in a significant association at *p* <.05 level; however, it should be noted that there was such an association at *p* <.10 level. Notably, incorporating the two dimensions of loneliness into model 3 meant that the coefficients for education and economic hardship were not significant, although living in a nursing home continued to play the same role. Neither of the models that included interactions between SES and loneliness (models 4 and 5) implied an improvement compared to model 3.

**Table 4:** Summary of logistic regression analyses to predict depression risk situation (potential case) from SES and emotional and social loneliness

	Model 1. Control variables		Model 2. Socioeconomic Status (SES)		Model 3. Loneliness		Model 4. Interaction SES and Loneliness (Education)		Model 5. Interaction SES and Loneliness (Economic Harships)		
	**B (SE)**	**Exp(B)**	**B (SE)**	**Exp(B)**	**B (SE)**	**Exp(B)**	**B (SE)**	**Exp(B)**	**B (SE)**	**Exp(B)**	
Sex (Female)	0.29(0.18)	1.335	0.09 (0.19)	1.101	0.17 (0.21)	1.192	0.20(0.21)	1.230	0.20(0.21)	1.230	
Age	0.01(0.01)	1.015	− 0.006 (0.01)	0.994	− 0.001 (0.01)	0.999	− 0.001(0.01)	0.999	− 0.001(0.01)	0.999	
No children	0.45(0.21)^*^	1.568	0.20 (0.23)	1.225	0.006 (0.25)	1.006	− 0.003(0.26)	0.997	− 0.003(0.26)	0.997	
*Limitation on ADL (reference: not limited)*											
Limited	1.35(0.19)^***^	3.865	1.26(0.19)^***^	3.526	1.11(0.21)^***^	3.033	1.16(0.22)^***^	3.195	1.16(0.22)***	3.204	
Strongly limited	2.16(0.27)^***^	8.689	1.97(0.28)^***^	7.168	1.78(0.31)^***^	5.941	1.85(0.31)^***^	6.406	1.85(0.31)***	6.408	
*Education (reference: incomplete primary)*											
Primary			− 0.33(0.21)	0.719	− 0.25 (0.24)	0.778	0.19(0.43)	1.212	0.19(0.43)	1.218	
Secondary			− 0.27(0.30)	0.759	− 0.03 (0.34)	0.967	0.43(0.54)	1.551	0.45(0.54)	1.579	
University			− 0.65(0.32)^**^	0.522	− 0.38 (0.35)	0.681	− 0.26(0.59)	0.767	− 0.23(0.60)	0.787	
Economich harships			0.62(0.19)^***^	1.861	0.39 (0.22)^†^	1.486	0.39(0.22)^†^	1.490	0.45(0.38)	1.578	
*Living arrangement (reference: living alone)*											
Living with partner			− 0.57(0.20)^**^	0.565	− 0.11 (0.23)	0.893	− 0.11(0.23)	0.891	− 0.11(0.23)	0.891	
Nursing home			0.71(0.24)^**^	2.035	0.62 (0.26)^*^	1.866	0.65(0.27) ^*^	1.921	0.65(0.27)^*^	1.918	
Loneliness (emotional)					1.05(0.11)^***^	2.874	1.20(0.23)^***^	3.346	1.23(0.26)^***^	3.434	
Loneliness (social)					0.17 (0.09)^†^	1.193	0.26(0.16)	1.306	0.26(0.18)	1.300	
*Interactions Education (reference: primary incomplete)*											
Primary*Social loneliness							− 0.12(0.21)	0.884	− 0.12(0.21)	0.884	
Secondary*Social loneliness							− 0.62(0.34) ^†^	0.537	− 0.62(0.35) ^†^	0.538	
University *Social loneliness							0.21(0.29)	1.234	0.21(0.30)	1.239	
Primary*Emotional loneliness							− 0.25(0.29)	0.773	− 0.26(0.29)	0.770	
Secondary*Emotional loneliness							0.11(0.39)	0.773	0.09(0.40)	1.105	
University*Emotional loneliness							− 0.18(0.40)	1.118	− 0.21(0.42)	0.809	
Economic harships* Social loneliness									0.01(0.20)	1.011	
Economic harships * Emotional loneliness									− 0.05(0.26)	0.943	
Constant	-3.24 (0.80)^***^	0.039	-1.16(0.98)	0.313	-2.93 (1.14)^***^	0.053	-3.30 (1.17) ^**^	0.830	-3.33(1.18)	0.036	
Cox & Snell R^2^	0.132^***^		0.174^***^		0.291^***^		0.296		0.296		
Nagelkerke R^2^	0.190^***^		0.251^***^		0.420^***^		0.428		0.428		

^***^p <.001; ^**^p <.01; ^*^p <.05; ^†^*p* <.10; Exp(B) = Odds Ratio; SE = Standard Error

Table 5 shows the results for calculating the models’ goodness-of-fit by replacing the quantitative variables of emotional and social loneliness with a variable that covers the potential identification of a case of loneliness. As described in the methodology section, a cut-off point of 3 was established. Table 5 hence reproduces the logic of the models defined for this study, making it possible to calculate the difference in the likelihood of identifying a case of depression among those people who are suffering potential situations of loneliness (or not). As might be expected, the results were consistent with those set out in Table 4. However, it should be noted that in model 3 the significant association between loneliness and depression entailed a difference in likelihood of over 300% compared to people who were not potentially in a position of loneliness. In addition, that model maintains a significant coefficient for the economic hardship measure, which increased the likelihood of depression by 51%. Finally, only the interaction between primary education and the potential presence of loneliness produced a significant coefficient (models 4 and 5), although it is necessary to take into account that its incorporation does not significantly improve explained variance.

**Table 5:** Summary of logistic regression analyses to predict depression risk situation (risk, potential case) from SES and risk of loneliness

	Model 1. Control variables		Model 2. Socioeconomic Status (SES)			Model 3. Loneliness			Model 4. Interaction SES and Loneliness (Education)			Model 5. Interaction SES and Loneliness (Economic Harships)			
	**B (DE)**	**Exp(B)**		**B (SE)**	**Exp(B)**		**B (SE)**	**Exp(B)**		**B (SE)**	**Exp(B)**		**B (SE)**	**Exp(B)**	
Sex (Female)	0.29(0.18)	1.335		0.09(0.19)	1.101		0.21(0.19)	1.238		0.20(0.20)	1.230		0.20(0.20)	1.231	
Age	0.01(0.01)	1.015		− 0.006(0.01)	0.994		0.001(0.01)	1.001		0.001(0.01)	1.001		0.001(0.01)	1.001	
No children	0.45(0.21)^*^	1.568		0.20(0.23)	1.225		0.01(0.24)	1.015		− 0.001(0.24)	0.999		− 0.004(0.24)	0.996	
*Limitation on ADL (reference: not limited)*															
Limited	1.35(0.19)^***^	3.865		1.26(0.19)^***^	3.526		1.14(0.20)^***^	3.142		1.17(0.20)^***^	3.244		1.17(0.20)^***^	3.239	
Strongly limited	2.16(0.27)^***^	8.689		1.97(0.28)^***^	7.168		1.81(0.29)^***^	6.154		1.87(0.30)^***^	6.515		1.87(0.30)^***^	6.517	
*Education (reference: incomplete primary)*															
Primary				− 0.33(0.21)	0.719		− 0.28(0.22)	0.754		0.07(0.28)	1.075		0.06(0.29)	1.072	
Secondary				− 0.27(0.30)	0.759		− 0.14(0.31)	0.864		0.18(0.38)	1.200		0.17(0.39)	1.188	
University				− 0.65(0.32)^**^	0.522		− 0.45(0.33)	0.634		− 0.25(0.40)	0.778		− 0.26(0.40)	0.769	
Economic harships				0.62(0.19)^***^	1.861		0.41(0.20)^*^	1.511		0.42(0.21)^*^	1.523		0.39(0.26)	1.478	
*Living arrangement (reference: living alone)*															
Living with partner				− 0.57(0.20)^**^	0.565		− 0.38(0.21) ^†^	0.679		− 0.40(0.21) ^†^	0.667		− 0.40(0.21) ^†^	0.668	
Nursing home				0.71(0.24)^**^	2.035		0.61(0.25)^*^	1.843		0.62(0.25)^*^	1.860		0.62(0.25)^*^	1.862	
Loneliness							1.40(0.20)^***^	4.083		2.02(0.37)^***^	7.584		1.98(0.42)^***^	7.310	
*Interactions Education (reference: primary incomplete)*															
Primary*Loneliness										− 0.95(0.47)^*^	0.386		− 0.94(0.47) ^*^	0.389	
Secondary*Loneliness										− 0.90(0.65)	0.404		− 0.89(0.65)	0.409	
University *Loneliness										− 0.48(0.69)	0.616		− 0.45(0.71)	0.636	
Economic harship* Loneliness													0.07(0.42)	1.081	
Constant	-3.24 (0.80)^***^	0.039		-1.16(0.98)	0.313		-2.19 (1.05)^*^	0.111		-2.39 (0.1.06)^*^	0.091		2.38(1.06)^†^	0.092	
Cox & Snell R^2^	0.132^***^			0.174^***^			0.224^***^			0.228			0.228		
Nagelkerke R^2^	0.190^***^			0.251^***^			0.323^***^			0.329			0.330		

^***^p <.001; ^**^p <.01; ^*^p <.05; ^†^*p* <.10; Exp(B) = Odds Ratio; SE = Standard Error

To confirm the robustness of the findings, the results obtained for model 3 were further validated by bootstrapping analysis (random sampling, *n* = 1.000). The results are shown in Table 6. For all the variables included in the model, the 95% bootstrap confidence intervals obtained in the analysis replicate the results obtained in the original analysis. Taken as a whole, these results suggest stability and consistency in the findings obtained in this study.

**Table 6:** Robustness checks for model 3. Bootstrap analysis (*n* = 1.000)

	Model: Social and Emotional Loneliness			Model: Loneliness (risk, cut-off point = 3)		
	**B (SE)**	**95% CI**		**B (SE)**	**95% CI**	
Sex (Female)	0.17(0.23)	− 0.27	0.64	0.21(0.21)	− 0.20	0.66
Age	− 0.001(0.01)	− 0.03	0.03	0.001(0.01)	− 0.02	0.02
No children	0.006(0.28)	− 0.63	0.50	0.01(0.26)	− 0.53	0.51
*Limitation on ADL (reference: not limited)*						
Limited	1.11(0.22)^***^	0.68	1.57	1.14(0.22)^***^	0.75	1.60
Strongly limited	1.78(0.33)^***^	1.18	2.48	1.81(0.32)^***^	1.21	2.48
*Education (reference: incomplete primary)*						
Primary	− 0.25(0.25)	− 0.74	0.24	− 0.28(0.23)	− 0.75	0.18
Secondary	− 0.03(0.36)	− 0.74	0.66	− 0.14(0.33)	− 0.83	0.48
University	− 0.38(0.36)	-1.15	0.31	− 0.45(0.34)	-1.19	0.19
Economich harships	0.39(0.23)	− 0.04	0.86	0.41(0.20)^*^	0.02	0.80
*Living arrangement (reference: living alone)*						
Living with partner	− 0.11(0.24)	− 0.57	0.36	− 0.38(0.22)	− 0.83	0.03
Nursing home	0.62(0.30)	0.05	1.25	0.61(0.28)^*^	0.07	1.20
Loneliness (emotional)	1.05(0.12)^***^	0.86	1.34			
Loneliness (social)	0.17(0.09)	− 0.02	0.37			
Loneliness (risk)				1.40(0.20)^***^	1.03	1.86
Constant	-2.93 (1.27)^*^	-5.75	− 0.57	-2.19 (1.09)^*^	-4.41	− 0.22

^***^p <.001; ^**^p <.01; ^*^p <.05

## Discussion

The results obtained in this study add empirical evidence for the association between SES and depression among older people. Both the bivariate analyses and models 1 and 2 of the logistic regression analysis suggest that low levels of education and the presence of economic hardship are associated with high scores for the depression measure [53, 54]. Tables 3 and 4 show that the incorporation of SES into model 2 of our logistic regression analyses resulted in statistically significant association coefficients, meaning that people with university qualifications showed a lower likelihood of being identified as potential cases of depression, while those experiencing economic hardships were in the opposite situation. There was an increase of 32% in explained variance for the outcome variable (depression) in model 2 compared to model 1 (the latter model only including the control variables, sex, age, children and limitations affecting mobility). This implies a moderate but significant predictive capacity of SES in terms of scores for depression.

However, the main contribution of this study was the analysis of the role played by loneliness in the association between SES and depression among older adults. In this regard, model 3 added the following to the logistic regression analysis: (a) scores for emotional and social loneliness, to predict the scores for the depression variable (Table 3); and (b) identification of a potential case of loneliness and its relationship to the existence of a potential case of depression (Table 4). These results specifically refer to the main research aim, which was to analyse the role played by loneliness in the relationship between SES and depression. First of all, the results shown in Table 3 indicated a significant association only between emotional loneliness and the existence of a potential case of depression, but not in the case of social loneliness. Moreover, and this is particularly significant, the incorporation of this association meant that the regression coefficients for level of education and economic hardship ceased to be significant. Similar results were obtained when introducing the loneliness variable into model 3 as a dichotomous variable (presence/absence) (Table 4). The only variation consisted of economic hardship maintaining a significant association with depression, although it is important to note that the intensity of that association was significantly reduced.

These results show that the relationship between socioeconomic status and depression among the older people who took part in the study was significantly affected by their experience of loneliness. In other words, the positive association between socioeconomic disadvantages and depression could arise indirectly as a result of its link to loneliness. Our results suggest that this is a plausible explanation, insofar as loneliness was significantly more prevalent in our sample among unqualified people than those with university qualifications. Moreover, loneliness occurred more frequently among participants who were experiencing economic hardship. These results coincide with those obtained in previous studies [35, 55, 56]. In this regard, socioeconomic difficulties can restrict relationships for older adults in terms of their social lives, limiting opportunities to take part in sociocultural activities and curtailing access to community resources that are valuable in combating loneliness [39], such that low SES during the ageing process can result in the truncation of social links [34]. It is even possible that low SES (meaning less education and more economic deprivation) may be related to living in contexts– whether neighbourhoods or nursing homes– offering social, economic, cultural and environmental resources that do not support people’s needs for socialisation and intimacy as they age. In other words, socioeconomic inequalities during ageing are reflected in the resources that comprise the place of residence, meaning that those in privileged social positions have greater enjoyment of age-friendly communities that “strive to find the best fit between the various needs and resources of older residents and those of the community” [57].These may include psychosocial resources, insofar as low income and lower levels of education can have an impact on self-esteem, self-efficacy and sense of mastery [17, 37]. These processes are closely related to the experience of emotional loneliness, on one hand, and depression, on the other [58].

In any case, rather than being confined to influencing the potential impact of SES, loneliness played a specific role in the results of this study (hence the 67% increase in explained variance when introducing this variable into our models compared to the model that only included SES). It is worth taking into account that recent studies have shown that the impact of socioeconomic inequalities on depression among older adults varies notably from country to country. Findings reported by Richardson et al. [59] using data corresponding to 18 countries in North America, South America, Europe, Asia, and the Middle East suggest considerable variability related to country-specific characteristics, such that they are more significant than any regional characteristics. In the European context, Sánchez-Moreno and Gallardo-Peralta [60] noted that one of the contextual factors with the greatest impact on the relationship between SES and depression is the level of perceived social support. In this regard, there were country-by-country variations in terms of the extent to which perceived social support was a protective factor against depression.

Along these lines, the link between loneliness and depression may be particularly significant in societies where accompaniment and support during difficult life situations falls essentially to support networks in general, and families in particular. This is true of Spain, where family support is a key cultural factor as a mechanism for social protection and psychosocial accompaniment [61]. Difficulties in generating intimate and reciprocal two-way relationships may increase the impact of loneliness on the deterioration of wellbeing during the ageing process [62]. At the same time, this relationship could be intensified in less resourceful contexts, in which SES (and particularly education and income) would be more significant in combating the negative effects of loneliness on depression [11]. In these contexts, people with more education and income would be less susceptible to the deleterious effects of loneliness.

The main limitation of this study concerns its cross-sectional design, which hinders an analysis of the impact of the variations of loneliness on depression, controlled by the dimensions of SES taken into account (education and economic hardship). In this context, moreover, the fact that loneliness levels can fluctuate over time needs to be taken into account. Awad et al. [63] suggest that this variation can occur on a weekly basis. This means that the specific time when the measurement is taken might not offer a general reflection of the experience of loneliness among interviewees. Finally, the loneliness scale used in this study is generally a reliable and valid measure across different countries. However, some still argue that gender and/or cultural differences may influence the response to the items of the scale [64].

## Conclusion

Alongside these limitations, this study suggests that loneliness– particularly emotional loneliness– can contribute to explaining the role played by socioeconomic inequalities in depression among older adults. Taking it into account when designing intervention plans could therefore contribute to reducing the social gradient in mental health for this group. In this regard, future research could further the understanding of the role that loneliness plays in the debate regarding social inequalities in health during the ageing process, and specifically the age-as-leveller hypothesis.

## Data Availability

The datasets generated and/or analysed during the current study are not publicly available at the moment due to temporary restrictions, but are available from the corresponding author on reasonable request.
